# A Complicated Case of a Paravalvular Leak Following Mitral Valve Replacement

**DOI:** 10.7759/cureus.11897

**Published:** 2020-12-04

**Authors:** Kashmala Khan, Pahnwat T Taweesedt, Sridhar Venkatachalam, Salim Surani

**Affiliations:** 1 Internal Medicine, Corpus Christi Medical Center, Corpus Christi, USA; 2 Cardiology, Corpus Christi Medical Center, Corpus Christi, USA; 3 Internal Medicine, University of North Texas, Dallas, USA

**Keywords:** paravalvular leak, mitral valve replacement, echocardiography - heart failure - valvular heart disease, aortic valve replacement, transthoracic echocardiogram, transesophageal echocardiogram, prosthetic heart valve, mitral regurgitation

## Abstract

Paravalvular leaks (PVL) are an uncommon sequelae of valve replacement but can be seen as a complication of both mechanic and prosthetic valves. Patients with PVL may remain asymptomatic or have detrimental complications, which include heart failure, hemodynamically significant hemolysis, and endocarditis. Management depends on the clinical presentation and the degree of the valvular leak. We hereby present a case report of a patient with a complicated clinical course secondary to a PVL of the mitral valve. The patient had a recent mitral valve replacement and presented with symptoms of heart failure that was refractory to standard medical therapy. Valvular abnormality was not seen with initial trans-thoracic echocardiogram (TTE), but with high clinical suspicion, a trans-esophageal echocardiogram (TEE) was done confirming a PVL. The patient’s condition continued to deteriorate; he became hemodynamically unstable with end-organ damage. Cardiothoracic surgery was consulted for surgical repair of the PVL. The patient, however, remained too unstable for surgery and the family opted for comfort measures per the patient’s wishes.

## Introduction

Patients with mitral or aortic valve replacements are at risk for developing several post-operative complications such as thromboembolism and hemorrhage. One such rare but potentially life-threatening complication is a paravalvular leak (PVL). A PVL occurs when the prosthetic valve’s ring fails to seal adequately or adhere to the native cardiac tissue. This causes an area of regurgitation or turbulent flow surrounding the valve [1]. PVL is an infrequent but serious complication following valve replacement. It is seen in 7% to 17% of patients following mitral valve replacement and in 2% to 10% after aortic valve replacement [2]. The imaging modality of choice to detect PVL include transthoracic echocardiogram (TTE), trans-esophageal echocardiogram (TEE) and cardiac computer tomography (CT). TTE is considered a first-line imaging modality to detect PVL. TEE is thought to provide more diagnostic value to detect PVL of the mitral valve than TTE. Suh et al. did find in a retrospective study that cardiac CT is comparable to TEE to detect PVL [2]. PVL can cause serious clinical outcomes in about 1% to 5% of patients which include symptomatic congestive heart failure, severe hemolytic anemia, and endocarditis [3]. Sampaio et al. demonstrated that endocarditis was the most common cause for reoperation regardless of the degree of PVL. They also noted that patients with severe PVL were most likely to undergo repeat surgery [4]. Here we present a case of a patient with a PVL who suffered a complicated clinical course.

## Case presentation

The patient is a 58-year-old male with a past medical history of mechanical aortic and mitral valve replacement (three months prior to presentation), paroxysmal atrial fibrillation status post left atrial appendage ligation (three months prior to admission), cardiac arrest (three months prior to admission), coronary artery disease status post percutaneous intervention to the mid-left circumflex artery (seven years prior to admission), paroxysmal ventricular tachycardia, and history of 15 pack-year smoking. The patient was scheduled for an outpatient thoracentesis for a left-sided pleural effusion, but was redirected to the emergency department (ED) because of severe dyspnea.

**Figure 1:**
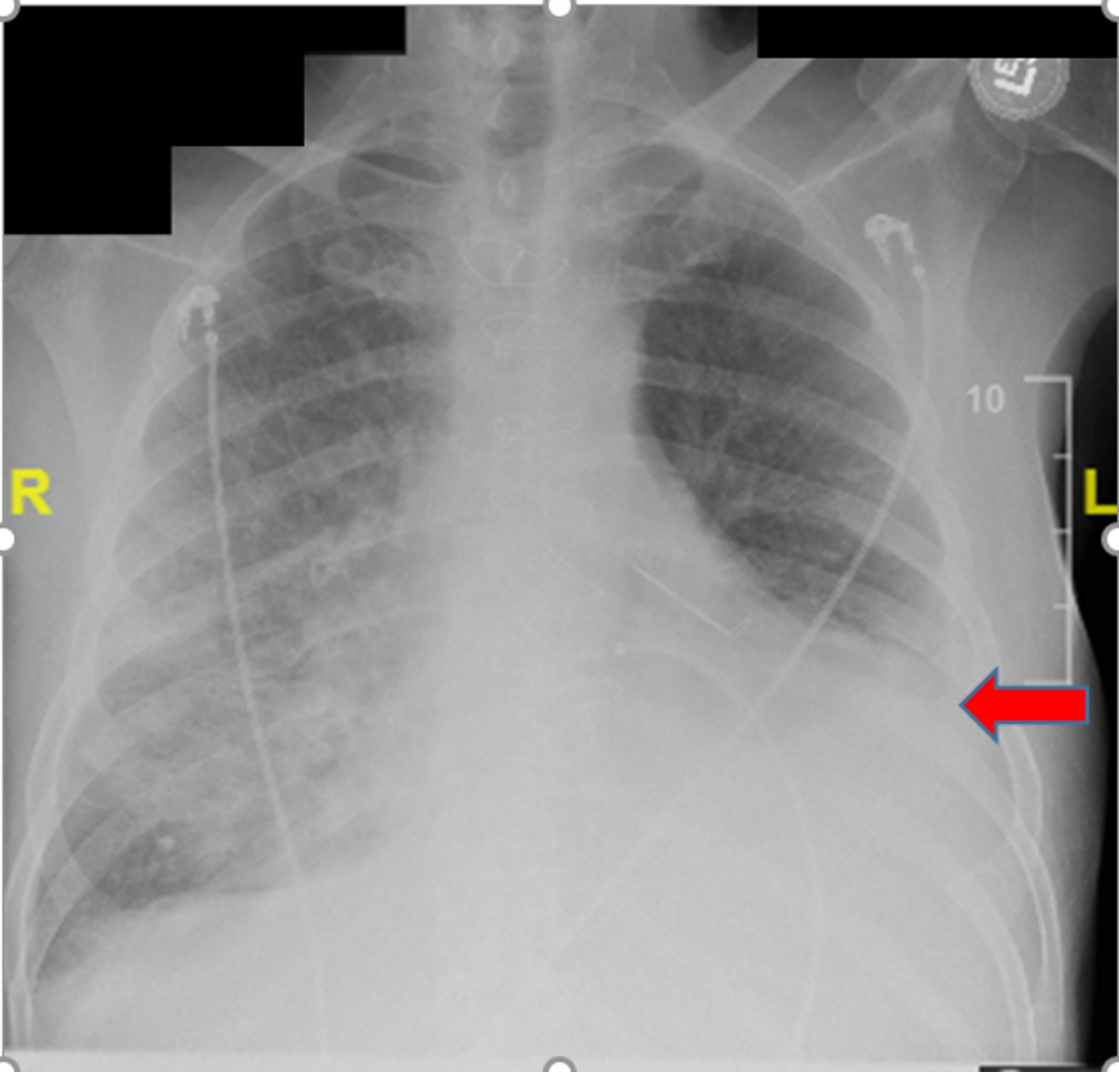
Initial chest X-ray with red arrow pointing at the left sided pleural effusion

In the ED, his blood pressure was 127/66 mmHg; he was found to be hypoxic on room air and initially placed on a non-rebreather mask. He was then placed on bilevel positive airway pressure (BiPAP) due to worsening dyspnea and respiratory effort. His initial arterial blood gas (ABG) on BiPAP showed a pH of 7.38, pCO2 of 33.7 L, pO2 195, and HCO3 of 19.6 L on 100% FiO2. The patient was on BiPAP for almost six hours but his symptoms worsened and he went into respiratory distress, finally requiring intubation. Per family, the patient had been having cough and shortness of breath over the last week prior to presentation but had denied having chest pain, abdominal pain, nausea, or vomiting. Of note, the patient had a recent hospitalization three months prior during which he had a mitral valve replacement for severe mitral regurgitation and a redo surgery due to a paravalvular leak, 10 days after the original surgery. A chest X-ray revealed cardiomegaly with congestive changes with bilateral pleural effusions (Figure 1).

Labs revealed a white cell count of 13.71 x 10³/uL, hemoglobin 9.7 mg/dL, platelet count 447,000/µL, lactic acid 5.6 mg/dL, international normalized ratio (INR) 2.4, potassium 4.3 mEq/L, creatinine 1.3 mg/dL, troponin 0.76 ng/ml, albumin 3.4 g/dL and amino-terminal pro-brain natriuretic peptide (NT-proBNP) level 5916 pg/ml.­­­ Electrocardiogram showed sinus tachycardia of 117 bpm without significant ST-T change from his baseline.

**Figure 2:**
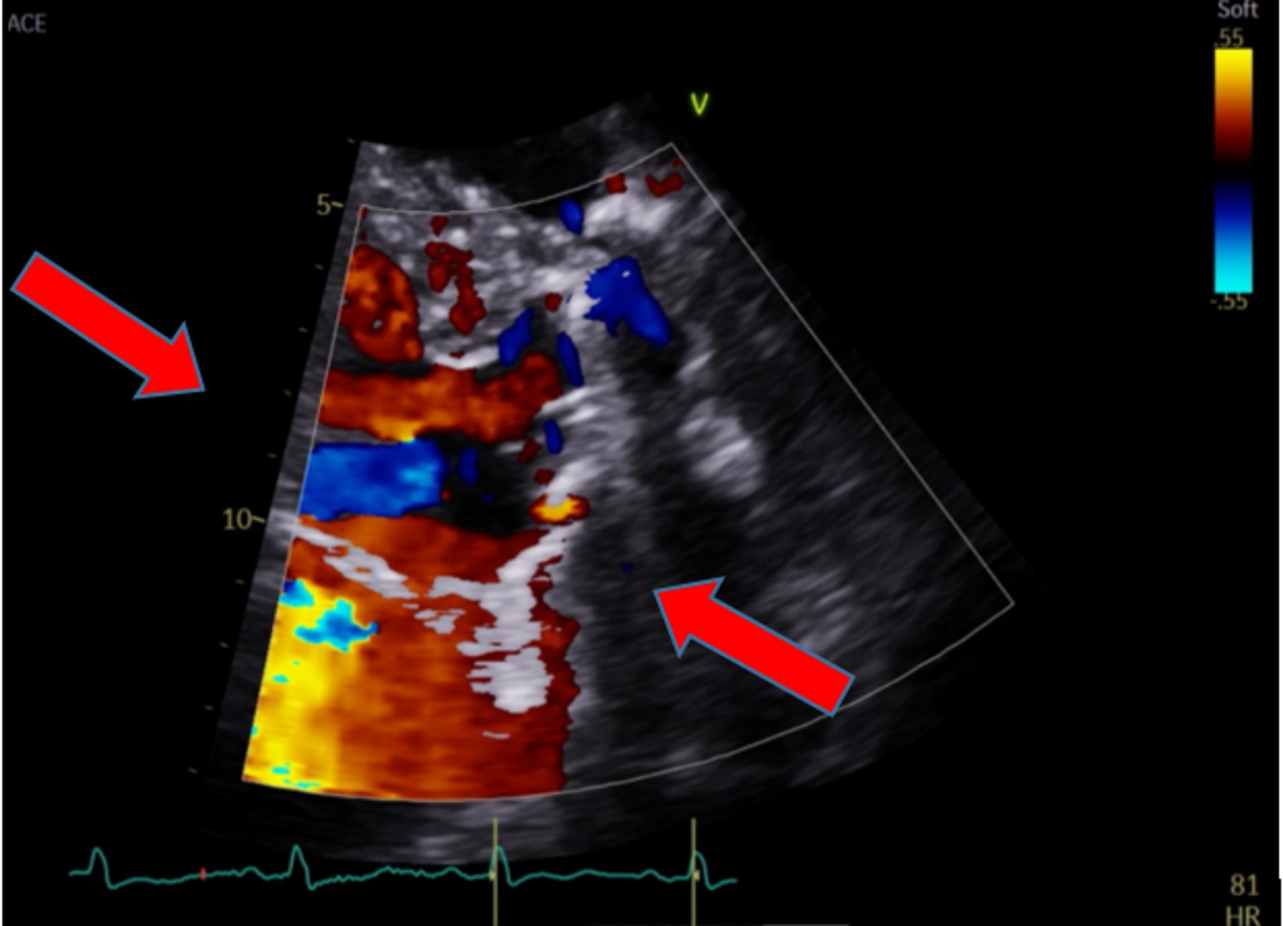
Transthoracic echocardiogram with suboptimal image quality and mild mitral regurgitation by color Doppler

**Figure 3:**
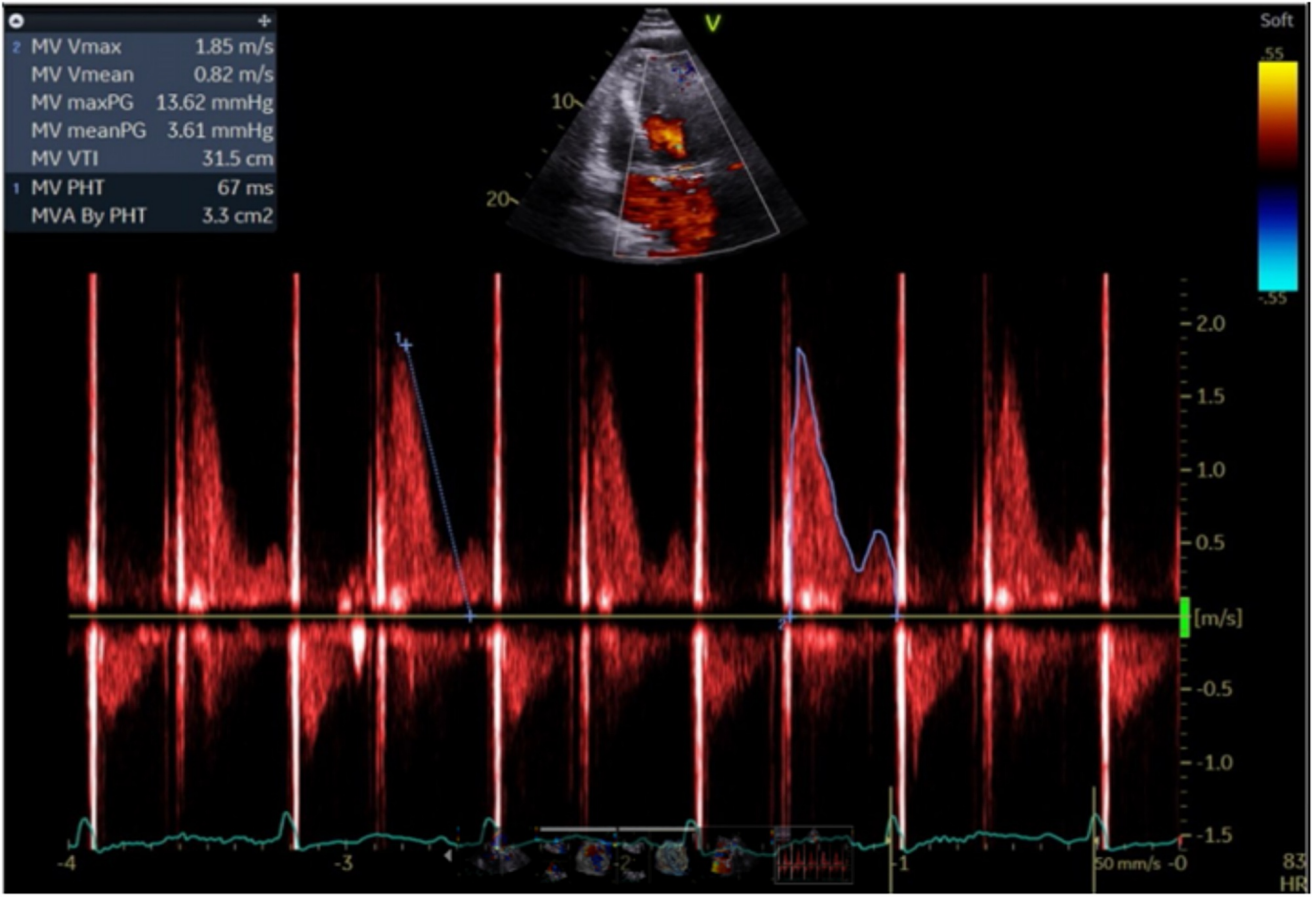
Mitral inflow velocity mapping on transthoracic echocardiogram without evidence of significant mitral regurgitation

The patient was admitted to the ICU for further management of acute pulmonary edema and respiratory failure. The patient’s respiratory status failed to improve. He was started on intravenous diuretic therapy with furosemide 40 mg twice daily and then switched to a furosemide drip. A TTE was technically insufficient to evaluate diastolic function but systolic function was preserved and ejection fraction was 60%-64%. There was a mechanic prosthetic aortic valve which was functioning normally, a mechanic prosthetic mitral valve which was also functioning normally. There was a loculated pericardial effusion measuring 4.1 cm in the inferolateral aspect of the left ventricle with no echocardiographic evidence of tamponade (Figures 2-3).

**Figure 4:**
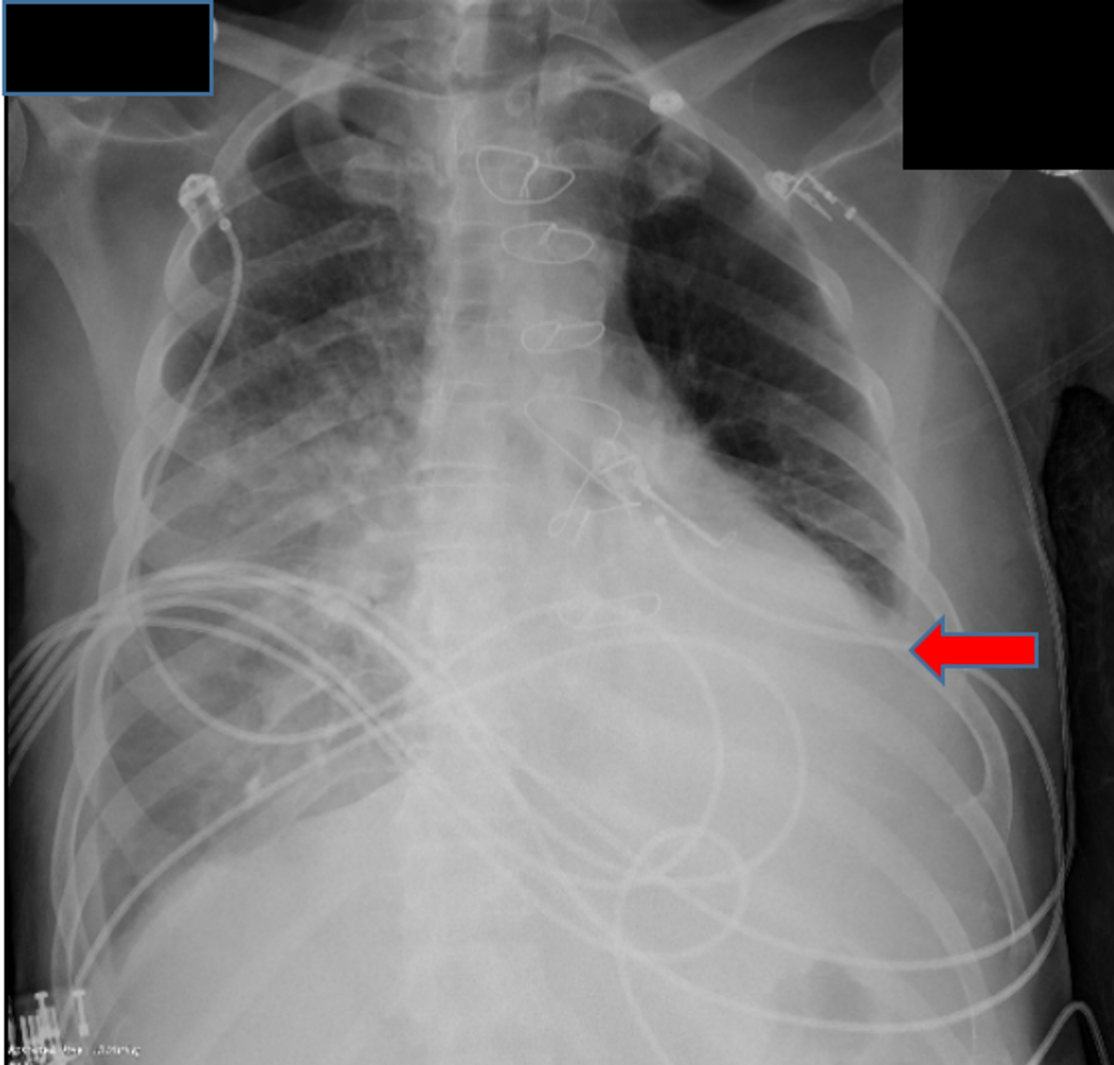
Subsequent chest X-ray showing small left sided pleural effusion (red arrow)

Due to the high transmission of severe acute respiratory syndrome coronavirus 2 (SARS-CoV-2) in our community, the patient was tested with reverse transcription-polymerase chain reaction (RT-PCR) test which was negative. His warfarin was discontinued and he was started on a heparin drip. The patient was successfully extubated the next day but required BiPAP alternating with a high flow nasal cannula due to hypoxia. His respiratory symptoms failed to improve with diuretic therapy. His pleural effusion was thought not to be large enough to warrant a thoracentesis (Figure 4).

**Figure 5:**
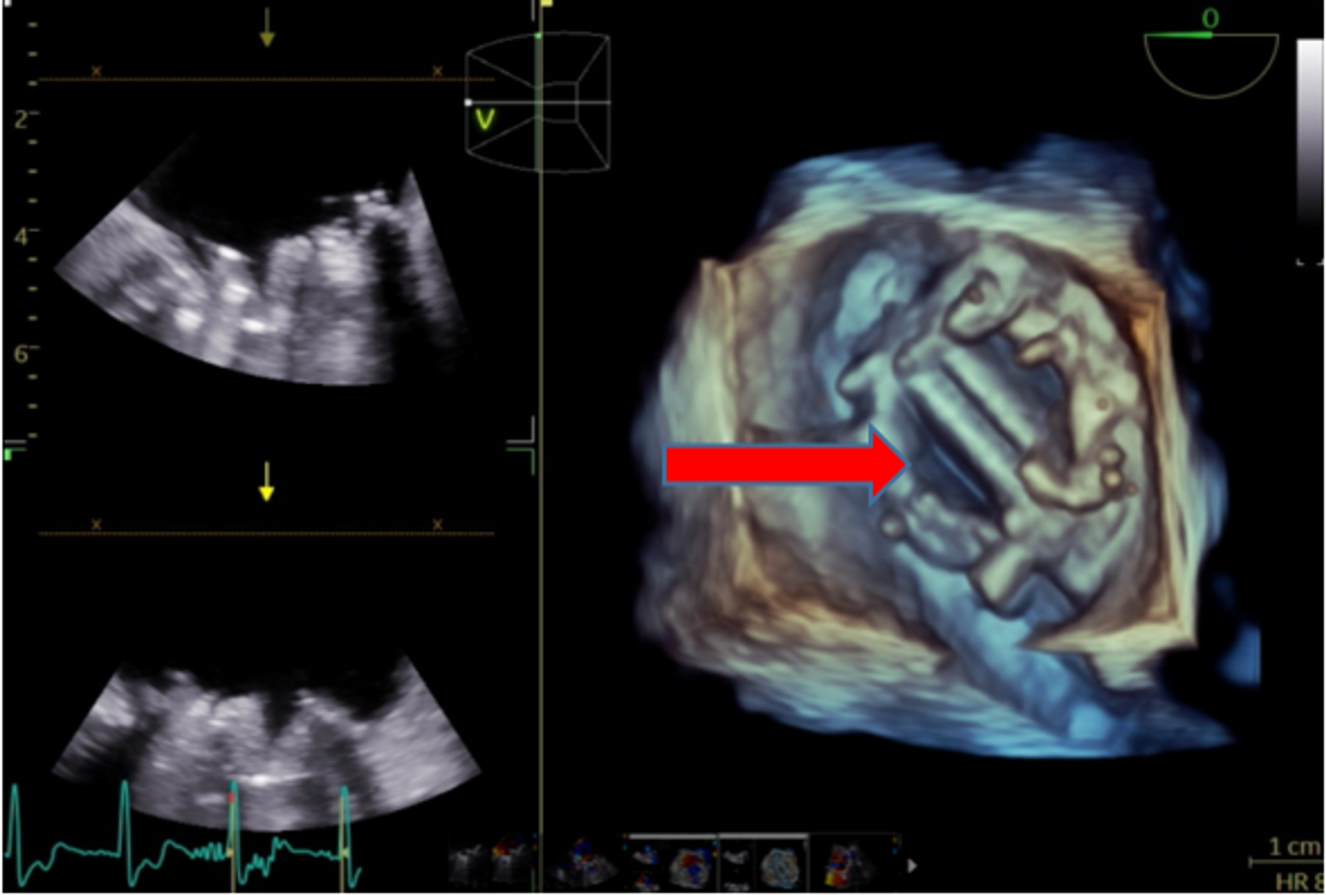
3D trans-esophageal echocardiogram of mitral valve and prosthesis (red arrow)

After failing to improve with medical management, a TEE was performed. The TEE showed a mitral valve with a bileaflet mechanical prosthesis in mitral position with normal leaflet motion (Figure 5).

**Figure 6:**
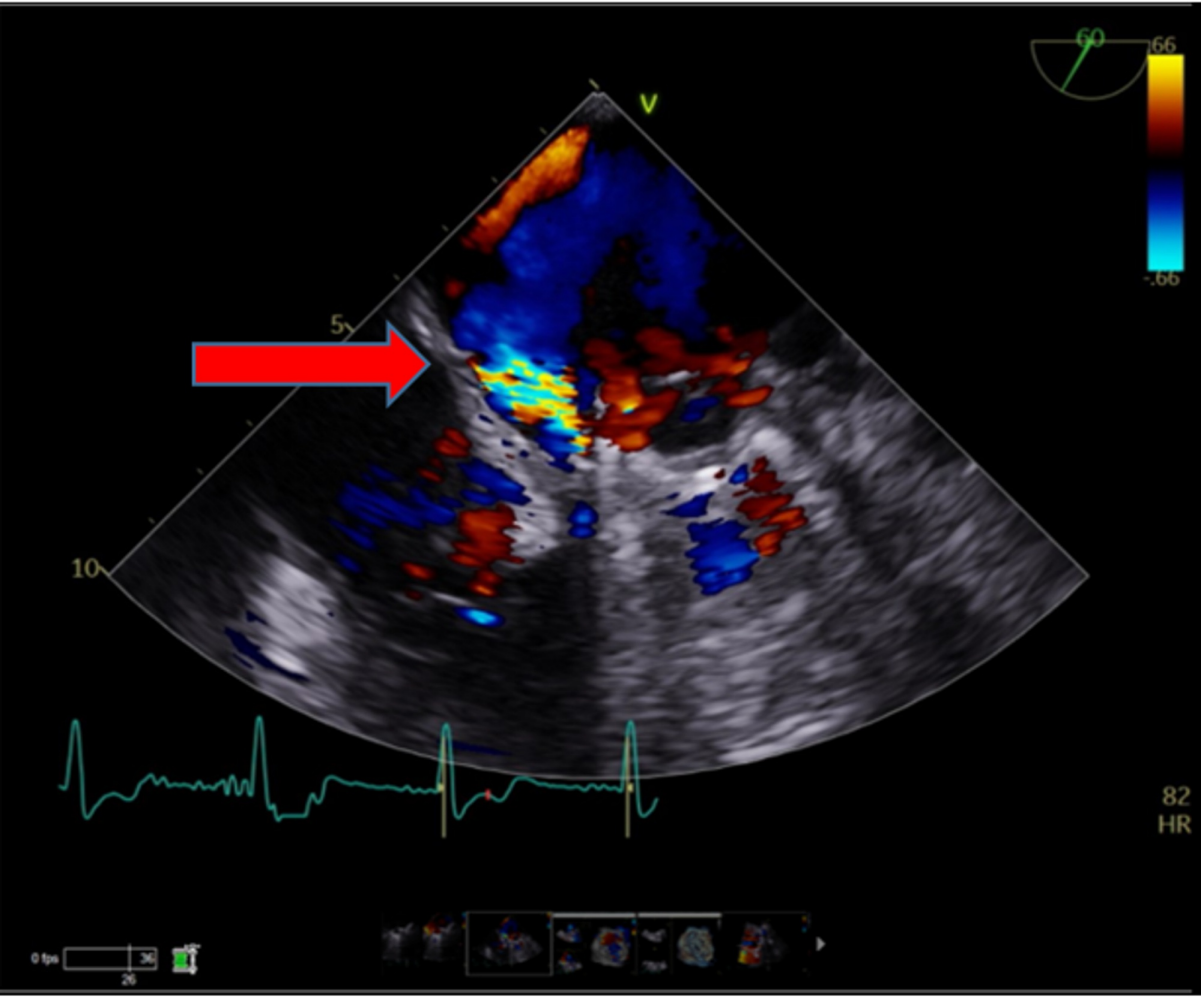
Mitral valve prosthesis with paravalvular regurgitant jet (red arrow) on trans-esophageal echocardiogram

**Figure 7:**
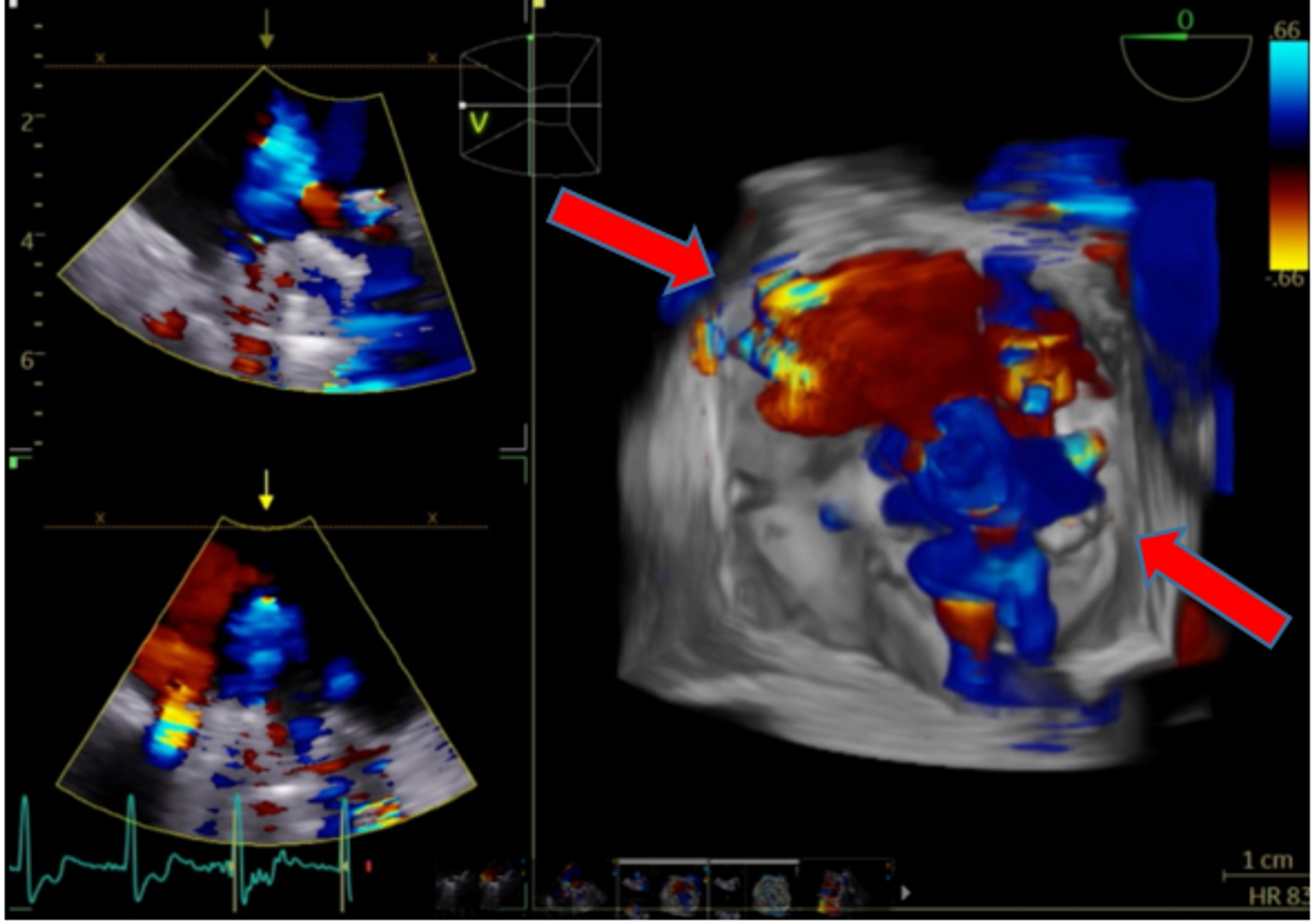
3D transesophageal echocardiogram of mitral valve showing multiple paravalvular regurgitant jets on color Doppler (red arrow)

A medial paravalvular leak was visualized with at least moderate mitral regurgitation (Figures 6-7).

There was a mechanical prosthetic valve in the aortic position, the valve was not well visualized. No evidence of regurgitation seen on color Doppler. The tricuspid and pulmonic valves were structurally normal with no evidence of regurgitation.

He was found to become hypotensive requiring vasopressor support. His respiratory function was worsening requiring reintubation on day 33 of hospitalization. There was concern for worsening paravalvular leak. The patient also developed acute kidney injury and severe metabolic acidosis requiring hemodialysis. The cardiovascular surgeon offered to do an open repair for the PVL, but the patient’s worsening condition would not permit it. His poor prognosis was discussed with the family and they decided to place the patient on comfort measures only.

## Discussion

PVL can have a very varied clinical course. About 40% of patients with PVL remain asymptomatic [5]. PVLs may initially present as a murmur, therefore a thorough physical examination is crucial. Laboratory testing can include assessing for hemolytic anemia and blood cultures for endocarditis [3]. The clinical presentation of a PVL may depend on the severity of regurgitation. Hemolytic anemia may be seen with small PVL, whereas large PVLs are more frequently associated with heart failure [5]. Our patient presented with heart failure which had no significant improvement after treatment with diuretics. To diagnose a PVL, the initial imaging of choice is a TTE. A TTE can provide information regarding the valve size, function, and has the ability to detect regurgitant jets through color Doppler flow. TTE can be limited due to the presence of acoustic shadows and artifacts [1]. Although a valuable imaging modality for identifying valvular abnormalities, TTE has a sensitivity of 57% and specificity of 63% only. In comparison, the sensitivity and specificity of a TEE are 86% and 88%, respectively. TEE adds value when there is high suspicion for a PVL [6]. TEE has been shown to be superior to TTE by detecting structural abnormalities, determining the degree of regurgitation and valve dysfunction [6].

The two treatment options for PVL are surgical correction and percutaneous trans-catheter closure. Surgery is preferred in patients who have large, severe PVL, failed conservative medical management, or present with refractory heart failure. Percutaneous closure is contraindicated in active endocarditis, intra-cardiac thrombi, and a large PVL involving more than one-third of the prosthetic annulus [7]. We were able to establish the significance of TEE and its diagnostic value in determining the presence of PVL. Cho et al. have demonstrated that patients with PVL following mitral valve replacement have worse clinical outcomes and prognosis when compared to aortic valve replacement [8].

As mentioned above, the initial TTE was unable to detect any PVL, but the patient’s worsening clinical status prompted further work up. The patient had symptoms of heart failure refractory to medical therapy for acute heart failure and a PVL was then confirmed on TEE. Surgery was the only option but prognosis was poor given that his clinical course was complicated with hemodynamic compromise and end-organ damage (cardiac, renal, and pulmonary). This can be explained by his significant comorbidities which include ischemic cardiomyopathy, previous cardiac arrest, and a history of arrhythmias. In addition, as mentioned above, a PVL followed by a mitral valve replacement has a worse prognosis and poor survival when compared to AVR. It is important to be vigilant when there is high clinical suspicion but TTE is negative for PVL.

## Conclusions

PVL is a well-known but infrequent complication of aortic and mitral valve replacement. It is important to recognize the signs and symptoms in such patients and be able to diagnose the condition in a timely manner. Imaging modalities used in the diagnosis of PVL include TTE, TEE, cardiac MRI, and cardiac CT. The most commonly used are TTE and TEE. As TTE can often miss the diagnosis of PVL, it is important to follow up with a TEE when the clinical suspicion is high.
